# Patient Satisfaction with Rural Medical Services: A Cross-Sectional Survey in 11 Western Provinces in China

**DOI:** 10.3390/ijerph16203968

**Published:** 2019-10-17

**Authors:** Jinlin Liu, Ying Mao

**Affiliations:** 1 Walter H. Shorenstein Asia-Pacific Research Center, Stanford University, Stanford, CA 94305, USA; 2 Research Center for the Belt and Road Health Policy and Health Technology Assessment, Xi’an Jiaotong University, Xi’an 710049, China; 3 School of Public Policy and Administration, Xi’an Jiaotong University, Xi’an 710049, China

**Keywords:** patient satisfaction, rural medical services, China

## Abstract

Rural medical services play an important role in protecting and promoting the health of the rural population; however, patient satisfaction with rural medical services has been understudied in China. A better understanding of the actual situation and the determinants involved will provide evidence for health-related policy makers and hospital managers to further improve rural medical services. A total of 9811 patients (5208 outpatients and 4603 inpatients) were included in this study from a cross-sectional survey conducted in rural hospitals from 11 western provinces in China. Three in five patients (including outpatients and inpatients) were satisfied with rural medical services. The mean overall satisfaction scores were 3.61 ± 0.857 and 3.80 ± 0.829 (out of a maximum of 5) for rural outpatients and inpatients, respectively. The most satisfying domains for outpatients and inpatients were medical service attitude and illness explanation, and waiting time and medical expenses were the domains that outpatients and inpatients were least satisfied with. Satisfaction with medical technology (OR: 1.73; 95% CI: 1.57–1.92) and satisfaction with trust in physicians (OR: 2.05; 95% CI: 1.85–2.28) were identified as the strongest predictors of outpatients’ and inpatients’ overall satisfaction with rural medical services, respectively. This study might shed light on rural medical services management in China.

## 1. Introduction

Health, the eternal topic of mankind, was put in the central position of the global Sustainable Development Goals (SDGs) by the United Nations in December 2015 [1]. Health systems and health services play a vital role in protecting and promoting human health. As the consumer of healthcare, a patient has an effective perspective to assess the quality of health services. In fact, patient satisfaction has been highlighted as an important indicator regarding health services outcomes. The World Health Organization (WHO) updated its global reference list of 100 core health indicators in 2018, in which patient satisfaction was included as one indictor to measure the quality and safety of care in health systems [2]. In addition, when U.S. News & World Report selects and ranks the best hospitals in the U.S., starting with 2019–2020 rankings, it includes the patient experience score based on patient satisfaction survey data [3]. Since 2019, in China, the General Office of State Council has underlined patient satisfaction as a key indicator of performance in the assessment of tertiary public hospitals [4]. The importance of patient satisfaction has reached a consensus worldwide.

Since the 1970s, the definitions and measures of patient satisfaction have attracted more attention. However, there is no consensus among the existing literature regarding the definition and measurement of patient satisfaction with regards to healthcare [5]. Both Risser and Greene et al. defined patient satisfaction as the perceived fulfillment of patients’ needs and desires through the real delivery of healthcare [6,7]. WHO [2], Ware et al. [8], Donabedian [9,10], and the Health Boards Executive (HeBE) in Ireland [11] have also put forward different definitions of patient satisfaction. A systematic review has summarized the domains used for patient satisfaction measurement in Saudi Arabia, which includes communication attributes, relational conduct, technical skill, personal qualities, availability, and accessibility [12]. Meanwhile, many empirical studies have been conducted to measure patient satisfaction in different countries, such as England [13], Pakistan [14,15], China [16,17,18,19,20], Germany [21], Saudi Arabia [22], the U.S. [23], Canada [24], Italy [25], and South Africa [26]. Among these studies, multifarious measures were employed and numerous determinants of patient satisfaction were identified. A systematic review conducted in 2017 summarized the determinants from 109 qualified international studies, which included healthcare provider-related determinants (e.g., technical care, interpersonal care, physical environment, access, organization characteristics, care continuity, and outcome) and patient-related characteristics (e.g., age, gender, education, socio-economic status, marital status, race, religion, geographic characteristics, visit regularity, length of stay, health status, personality, and expectations) [27]; however, how these determinants influence patient satisfaction is still inconclusive and inconsistent among different studies.

Currently, rural health systems are still relatively weaker than urban health systems worldwide, as the well-functioning health system is built on having a qualified and sufficient health workforce and sufficient infrastructures, medicines, technologies, etc. [28]. Although the world is undergoing the largest wave of urban growth, approximately 44.7% of the global population still lived in rural areas in 2018 [29], hence the urgent need for improving rural health systems. In addition, a better understanding of patient satisfaction with rural medical services and potential determinants would help policy- and decision-makers to implement programs better tailored to rural patients’ health needs and desires and further improve rural health systems [30,31]. Some evidence has been found about patient satisfaction with rural medical services in Italy [25], South Africa [26], India [32,33], Rwanda [34], Bangladesh [35], Latin America [36], and the U.S. [37]. In China, one of the five key objectives of China’s new healthcare reform, launched in 2009, is to strengthen the basic-level health service delivery system, especially the rural health system. However, very few related studies have been found in China. Gu et al. reported that rural patient satisfaction with the New Cooperative Medical Scheme (NCMS) was influenced by the NCMS’s reputed reliability, value, and convenience [38]. Liu et al. found the optimized health service delivery in less-developed rural areas could improve patient satisfaction with care [39], specifically, the satisfaction scores in the intervention county respondents increased from 21.4 (95% CI 21.1–21.7) to 22.1 (95% CI 21.7–22.4) with no change in the comparison area. Yan et al. identified perceived convenience, being enrolled in the NCMS, age, and the income of patients as the significant influencing factors associated with their satisfaction with rural medical services [40]. Patient satisfaction with rural general medical services has been understudied in China.

Based on the above, using data extracted from a cross-sectional survey among patients (i.e., outpatients and inpatients) seeking medical services in rural areas in 11 western provinces of China, this study aimed to analyze patient satisfaction with rural medical services in China and identify the related determinants.

## 2. Materials and Methods

### 2.1. Study Design and Participants

**Figure 1:**
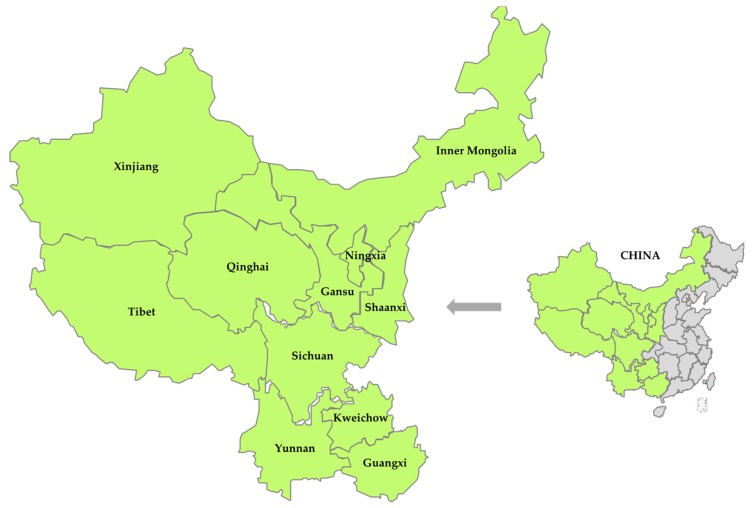
Sample provinces in western China.

As Figure 1 shows, this cross-sectional study was carried out in 11 western provinces in China, including Gansu, Guangxi, Kweichow, Inner Mongolia, Ningxia, Qinghai, Shaanxi, Sichuan, Tibet, Xinjiang, and Yunnan. A total of 11 research teams from the 11 provinces participated in the survey coordinated by Xi’an Jiaotong University in Shaanxi Province.

**Figure 2:**
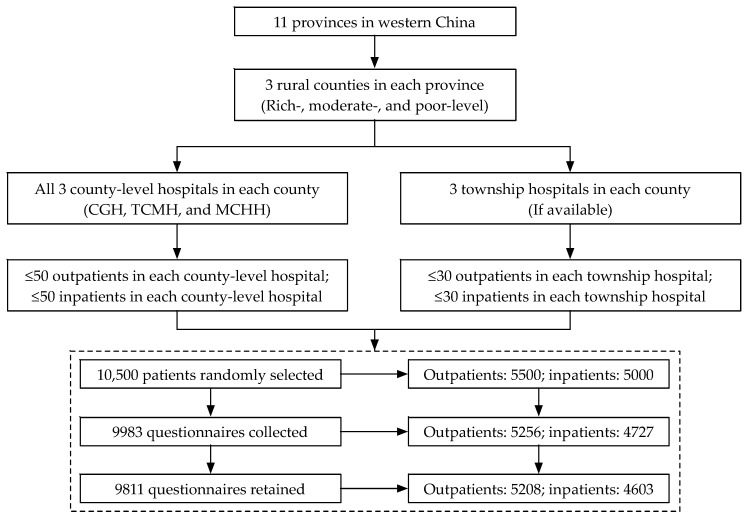
Study profile. CGH: county general hospital; TCMH: traditional Chinese medicine hospital; MCHH: maternal and child health hospital.

A three-stage random sampling method was used. As Figure 2 shows, first, three rural counties (rich, moderate, and poor) were randomly selected according to the stratification criteria of the economic development level of all the rural counties in each province individually. Second, in China, every county has three county-level hospitals, including one county general hospital (CGH), one traditional Chinese medicine hospital (TCMH), and one maternity and child health hospital (MCHH), that can provide medical services for residents; additionally, there are several township hospitals (THs) in a county. All three county-level hospitals and three township hospitals (if available), selected randomly in each county, were invited to participate in our survey. Third, ≤50 outpatients and ≤50 inpatients in each of the three county-level hospitals and ≤30 outpatients and ≤30 inpatients in each township hospital were selected randomly. The sample sizes for patients, i.e., inpatients and outpatients, were discussed and determined by all the co-PIs (Principal Investigators) from the 11 research teams and experts from WHO under comprehensive consideration of the study objective, survey duration, and budget.

Approximately 5500 outpatients and 5000 inpatients in total were randomly selected; however, only the patients who were willing to participate in the survey answered the questionnaires. A total of 9983 questionnaires, including 5256 from outpatients and 4727 from inpatients, were collected. The response rates for the outpatients and inpatients were 95.6% and 94.5%, respectively. Of which, 5208 (99.1%) and 4603 (97.4%) questionnaires for outpatients and inpatients were retained in the study after the data were incorporated and checked by Xi’an Jiaotong University. A total of 172 questionnaires (48 for outpatients and 124 for inpatients) were excluded due to missing values regarding overall satisfaction with rural medical services.

### 2.2. Data and Variables

A brief questionnaire was developed for data collection, which referred to the questionnaires of the China National Health Service Surveys (CNHSSs). The CNHSSs have been conducted by the National Health Commission of China (formerly known as the Ministry of Health) every five years since 1993. All the co-PIs and experts from WHO participated in the design of the questionnaire. Small-scale pre-surveys were conducted among patients to validate the logic, rationality, etc., of the questionnaire. After the questionnaire had been finalized, the survey was conducted in 11 western provinces simultaneously to collect data from June to December 2013. All the questionnaires were completed by the investigators through face-to-face interviews with patients. Considering the objectives of this study, only relevant variables and data were extracted from the whole dataset, which consisted of two sections: (1) general sociodemographic characteristics and (2) satisfaction with rural medical services, including outpatient and inpatient services.

The variables used to measure the sociodemographic characteristics of outpatients and inpatients were (1) gender: female and male; (2) age was a continuous variable and was divided into three groups: ≤30 years, 31–45 years, and ≥46 years; (3) education: illiteracy, primary school, junior high school, and senior high school or above; (4) occupation: farmer and nonfarmer; (5) average monthly income over the past year: no regular income, ≤2000 Yuan, and ≥2001 Yuan; and (6) type of hospital: TH, MCHH, TCMH, and CGH.

In terms of patient satisfaction with rural medical services, Likert five-point scales were developed. Ten variables were used to measure the outpatient satisfaction with rural medical services, namely satisfaction with travel time, waiting time, illness explanation, treatment consultation, medical technology, medical service attitude of health worker, medical facility, hospital environment, medical expenses, and overall medical service satisfaction. The nine variables used to measure inpatient satisfaction with rural medical services were illness explanation, treatment consultation, medical technology, medical service attitude, trust in physicians, medical facility, hospital environment, medical expenses, and overall satisfaction. Each variable was given a statement with the options “strongly dissatisfied”, “dissatisfied”, “neither dissatisfied nor satisfied”, “satisfied”, or “strongly satisfied”; the options were assigned a score of 1, 2, 3, 4, and 5, respectively.

### 2.3. Statistical Methods

The continuous variable in the study, i.e., age, was tested for normality first with the one-sample Kolmogorov–Smirnov test. The original variable of age presented an abnormal distribution after the test and was described using the “median” and “interquartile range (IQR)”. The categorical variables were described using “number” and “percentage”. Meanwhile, the variables related to patient satisfaction with rural medical services were also displayed by “mean” score and “standard deviation (SD)”.

The internal consistency and reliability of the questionnaires in terms of outpatient and inpatient satisfaction with rural medical services were tested using Cronbach’s α. A one-way ANOVA was conducted to assess the difference in the mean score of overall satisfaction with rural medical services among outpatients and inpatients in different groups with different sociodemographic characteristics. Spearman correlation analyses were used to identify the correlation between the overall satisfaction score for rural medical services and the satisfaction score for each indicator.

Multiple linear regression (MLR) and binary logistic regression (BLR) were both conducted to identify the influencing factors associated with overall outpatient and inpatient satisfaction with rural medical services. When conducting the MLR analyses, the overall satisfaction scores of outpatients and inpatients were set as the dependent variables, all the specific indicators related to satisfaction with rural medical services were set as the independent variables, and all the sociodemographic characteristics of patients were set as the confounders. In the BLR analyses, the dependent variables, i.e., outpatients’ and inpatients’ overall satisfaction, were set as the binary variables; specifically, “strongly satisfied” and “satisfied” were defined as “positive answers” (1 = satisfied) and “neither dissatisfied nor satisfied”, “dissatisfied”, and “strongly dissatisfied” were “negative answers” (0 = dissatisfied). Furthermore, the independent variables and confounders were similar to those in the MLR analyses. In addition, the independent variables and confounders introduced into the MLR and BLR analyses were those that had been significant in the univariate analyses, i.e., one-way ANOVA and Spearman correlation analyses. The analyses for outpatients and inpatients were independent of each other. All the significance levels were set at *p*-value < 0.05.

The Statistical Package for Social Sciences 24.0 (SPSS, IBM, Armonk, NY USA) for MAC was used for data analysis.

### 2.4. Ethics

This study was approved by the Ethics Committee of the School of Medicine of Xi’an Jiaotong University (China), and the approval number was 2014189. The questionnaire was anonymous and verbal informed consent was obtained from all participating patients. The data used during the current study are available from the corresponding author on reasonable request.

## 3. Results

### 3.1. Sociodemographic Characteristics

The Cronbach’s α of the questionnaires were 0.769 for outpatients and 0.804 for inpatients. As both values were >0.7, this is a positive indicator of internal consistency and the acceptable reliability of the questionnaires [16]. A total of 5208 outpatients and 4603 inpatients were included in this study. The outpatients’ and inpatients’ sociodemographic characteristics are summarized in Table 1.

In terms of the outpatients, 57.0% were female. The median age was 36 years (IQR: 27–48 years), and 70.1% were younger than 45 years. A total of 67.8% attained the education of junior high school or below, and 42.2% of the outpatients were farmers. A total of 25.7% had no regular income per month over the past year, and only 28.7% had an average monthly income of more than 2001 Yuan over the last year before the survey. In addition, 39.2% of the outpatients were patients of township hospitals, and 27.6% were patients of county general hospitals.

In terms of the inpatients, 55.8% were female. The median age was 42 years (IQR: 28–58 years), and 56.3% were younger than 45 years. A total of 62.1% attained the education of junior high school or below, and 49.0% of the inpatients were farmers. A total of 32.9% had no regular income per month, and only 25.2% of inpatients had a monthly income of more than 2001 Yuan. In addition, 34.2% of the inpatients were patients of township hospitals, and 32.3% were patients of county general hospitals.

### 3.2. Patient Satisfaction with Rural Medical Services

Table 2 and Table 3 show the results of outpatient satisfaction and inpatient satisfaction with rural medical services, respectively. A total of 60.2% of outpatients and 70.9% of inpatients were satisfied (i.e., satisfied or strongly satisfied) overall with rural medical services. The mean overall satisfaction scores were 3.61 ± 0.857 and 3.80 ± 0.829 out of a maximum of 5 for outpatients and inpatients, respectively. Medical service attitude was the most satisfying domain for outpatients, whereas it was illness explanation for inpatients. Waiting time and medical expenses were the domains that outpatients and inpatients were least satisfied with. Each of the outpatient satisfaction indicators and inpatient satisfaction indicators scored >3.00. Meanwhile, the outpatients’ overall satisfaction with rural medical services was significantly correlated with each of the nine satisfaction indicators (i.e., travel time, waiting time, etc.), having the highest Spearman correlation with “satisfaction with medical technology” (r = 0.376, *p* < 0.001) and the least correlation with “satisfaction with travel time” (r = 0.121, *p* < 0.001). In addition, inpatients’ overall satisfaction was significantly correlated with all the eight satisfaction indicators, having the highest Spearman correlation with “satisfaction with trust in physicians” (r = 0.463, *p* < 0.001) and the least with “satisfaction with medical expenses” (r = 0.205, *p* < 0.001).

### 3.3. Influencing Factors of Patients’Overall Stisfaction with Rural Medical Services

The results based on the one-way ANOVA in Table 1 show that age (*p* < 0.001), occupation (*p* < 0.01), income (*p* < 0.01), and type of hospital (*p* < 0.01) were significantly correlated with the outpatients’ overall satisfaction with rural medical services. Specifically, outpatients who reported a significantly higher score of overall satisfaction were those who were 46 years and older, those who were farmers, those who received an income of ≥2001 Yuan per month, and those who were seeking medical services in MCHHs. Additionally, Table 1 shows that age (*p* = 0.001) and type of hospital (*p* < 0.001) were significantly correlated with the inpatients’ overall satisfaction of rural medical services. Specifically, inpatients who reported a significantly higher score of overall satisfaction were those who were 46 years and older and those who were seeking medical services in the MCHHs.

In addition, the results of the Spearman correlation analyses in Table 2 show that significant positive correlations were observed between the outpatients’ overall satisfaction with rural medical services and all the nine satisfaction indicators (*p* < 0.001), i.e., the higher the score of each of the nine satisfaction indictors, the higher the outpatients’ overall satisfaction score. Meanwhile, Table 3 shows that inpatients’ overall satisfaction had significant positive correlations with all the eight satisfaction indicators (*p* < 0.001).

Multivariate analyses were conducted using MLRs and BLRs to further identify the influencing factors of patient satisfaction with rural medical services. Table 4 shows the results of the influencing factors associated with the outpatients’ overall satisfaction with rural medical services. The results of the MLRs were consistent with those of the BLRs and show that outpatients’ overall satisfaction was significantly associated with satisfaction with travel time, waiting time, illness explanation, medical technology, medical service attitude, medical facility, hospital environment, medical expenses, age, income, and type of hospital. Taking the BLR results as an explanation, a high satisfaction with travel time (OR: 1.18; 95% CI: 1.09–1.28), waiting time (OR: 1.15; 95% CI: 1.07–1.25), illness explanation (OR: 1.23; 95% CI: 1.11–1.36), medical technology (OR: 1.73; 95% CI: 1.57–1.92), medical service attitude (OR: 1.51; 95% CI: 1.38–1.65), medical facility (OR: 1.27; 95% CI: 1.15–1.41), hospital environment (OR: 1.23; 95% CI: 1.11–1.36), and medical expenses (OR: 1.15; 95% CI: 1.06–1.25) would significantly increase the outpatients’ overall satisfaction with rural medical services; meanwhile, the outpatients who were 46 years or older (OR: 1.26; 95% CI: 1.06–1.49, compared with “≤30 years”) or received an income of 2001 Yuan or more per month (OR: 1.25; 95% CI: 1.03–1.51, compared with “no regular income”) had significantly higher overall satisfaction with rural medical services. However, outpatients who were seeking medical services in CGHs had significantly lower overall satisfaction (OR: 0.75; 95% CI: 0.64–0.89) compared with those in THs.

Table 5 shows the results of influencing factors associated with the inpatients’ overall satisfaction with rural medical services. The results of the MLRs and the BLRs indicate that satisfaction with illness explanation, medical technology, medical service attitude, trust in physicians, hospital environment, medical expenses, age, and type of hospital were significantly associated with the inpatients’ overall satisfaction with rural medical services. Taking the BLR results as an explanation, a high satisfaction with illness explanation (OR: 1.62; 95% CI: 1.43–1.85), medical technology (OR: 1.43; 95% CI: 1.26–1.62), medical service attitude (OR: 1.81; 95% CI: 1.65–1.98), trust in physicians (OR: 2.05; 95% CI: 1.85–2.28), hospital environment (OR: 1.50; 95% CI: 1.33–1.69), and medical expenses (OR: 1.56; 95% CI: 1.41–1.73) would significantly increase the inpatients’ overall satisfaction with rural medical services; meanwhile, the inpatients who were 31 years or older (for “31–45 years”, OR: 1.25 and 95% CI: 1.01–1.55; for “≥46 years”, OR: 1.39 and 95% CI: 1.13–1.70, compared with “≤30 years”) or who were seeking medical services in MCHHs (OR: 1.67; 95% CI: 1.28–2.19, compared with “THs”), TCMHs (OR: 1.95; 95% CI: 1.52–2.51), and CGHs (OR: 1.33; 95% CI: 1.09–1.63) had significantly higher overall satisfaction with rural medical services.

## 4. Discussion

As far as we know, this is the first study to analyze patient satisfaction with rural medical services and identify related determinants based on such a large-scale cross-sectional survey, covering 11 provinces in western China. A total of 9811 patients, including 5208 outpatients and 4603 inpatients, participated in the study. It provides relevant evidence and experience from China for the growing body of research on rural health systems from the perspective of patient satisfaction.

The results showed that about three in five patients were satisfied with rural medical services; specifically, 60.2% of outpatients and 70.9% of inpatients were satisfied or very satisfied. These results are consistent with previous studies. For example, Boovaragasamy et al. reported that ≥64.5% of 584 patients were satisfied with rural healthcare and the medical infrastructure facilities in Puducherry [32]. Additionally, Mutaganzwa et al. found that ≥50% of patients were satisfied with the antenatal care and maternity services in rural Rwanda [34], and 53.2% of young adult, rural Latinos were satisfied with the healthcare services they had used previously [36]. The results indicate that rural health systems are improving continuously and that some positive effects were achieved, such as the good patient satisfaction with rural medical services. However, attention should be paid to domains of rural medical services with low patient satisfaction scores.

Our study found that waiting time was identified as the domain with the lowest satisfaction score (3.16 ± 0.941) among rural outpatients, and it was also identified as an influencing factor associated with the outpatients’ overall satisfaction with rural medical services. A high satisfaction with waiting time (i.e., short waiting time) would bring a high overall satisfaction among outpatients for rural medical services (OR: 1.15; 95% CI: 1.07–1.25). The findings are very consistent with results in previous studies. Sun et al. pointed out that outpatients were most dissatisfied with the waiting time for tertiary healthcare in China [16]. In a Pakistani study, half of the patients receiving community pharmacy services were dissatisfied with the waiting time [15]. A systematic review, conducted in 2017, based on international evidence indicated that a shorter waiting time was positively associated with patient satisfaction [27]. The latest evidence from Godley et al., who conducted a pre/postintervention study, revealed that a decrease in waiting times was associated with a significant increase in the patient satisfaction score [41]. Moreover, satisfaction with the travel time to hospital was also identified to be significantly associated with the outpatients’ overall satisfaction with rural medical services (OR: 1.18; 95% CI: 1.09–1.28), which is in accordance with existing evidence [12,21,27,42]. There is no doubt that a longer travel time or a longer waiting time would bring inconvenience for patients when accessing medical services and have a negative impact on their satisfaction. This study highlights the importance of medical service accessibility.

Medical expense was also detected as a domain with patients’ low satisfaction scores, with the lowest satisfaction score being among rural inpatients (3.16 ± 0.887). Enakshi et al. found that 60.2% of patients were unsatisfied with the cost of rural healthcare in Southern India [33]. Consistently with earlier studies [16,18,27,42,43,44], our study indicates that a low satisfaction with medical expenses would significantly result in low patients’ overall satisfaction with rural medical services. The hospital and treatment costs for inpatients and outpatients have been rising both in urban and rural areas of China. According to the China Health Statistical Yearbook 2018, the average outpatient service expense each time, among all hospitals in China, increased from 166.8 Yuan in 2010 to 257.0 Yuan in 2017, and the average inpatient service expense per capita among all hospitals in China increased from 6193.9 Yuan in 2010 to 8890.7 Yuan in 2017 [45]. Unreasonable and excessive medical services still exist in China, and despite the rapid improvement of health insurance systems, the out-of-pocket payments still impose a great financial challenge to patients [18], especially for patients living in rural areas where the income level of residents is much lower than their counterparts in urban areas. Therefore, the affordability of medical services remains challenging for rural residents.

This study indicates that outpatient satisfaction with medical technology was the strongest influencing factor associated with the overall satisfaction with rural medical services (OR: 1.73; 95% CI: 1.57–1.92). The mean outpatient satisfaction score on medical technology was 3.53. Meanwhile, it was also significantly associated with inpatients’ overall satisfaction (OR: 1.43; 95% CI: 1.26–1.62). As an indicator of technical care, medical technology is closely related to medical service quality. Wu et al. found that patients who were satisfied with the medical skill level were 1.31 times more likely to be satisfied with the overall community health service in Shenzhen of China [42]. A similar finding has been reported in Saudi Arabia that good medical technology would bring about good patient satisfaction [12]. In addition, satisfaction with the medical facility was significantly associated with the outpatients’ overall satisfaction with rural medical services (OR: 1.27; 95% CI: 1.15–1.41). Medical facility, like medical technology, is another vital technical care indicator. Both the studies from Wu et al. [42] and Chimbindi et al. [26] have reported similar results. However, medical facility was not a significant factor associated with inpatients’ overall satisfaction in our study, which is consistent with the study by Hussain et al. conducted in Pakistan [14].

Satisfaction with trust in physicians was identified as the strongest influencing factor related to the inpatients’ overall satisfaction with rural medical services (OR: 2.05; 95% CI: 1.85–2.28), which implies that building a better patient–physician relationship will contribute to improving inpatients’ overall satisfaction to a large extent. Sun et al. reported similar results with our study in that the dimension of the patient–doctor relationship (i.e., trust in medical staff) was the strongest predictor of the patients’ overall satisfaction with tertiary healthcare in China [16]. The results of our study are also consistent with many previous studies conducted in Germany [21], China [43,46,47], and the U.S. [48]. Better trust in physicians would definitely help patients better cooperate with physicians’ treatment and would contribute to better medical service outcomes; additionally, studies have shown that, in this case, patients are more likely to recommend physicians to other people [21,48].

Our study indicates that the interpersonal care of medical service providers was significantly associated with the patients’ (i.e., outpatients and inpatients) overall satisfaction with rural medical services. Specifically, the patients’ satisfaction with medical service attitude or with illness explanation would significantly improve their overall satisfaction with rural medical services. Patients could feel respect, politeness, etc. from positive medical service attitudes of health workers. Some studies found that the medical service providers’ affective behaviors, especially respect and politeness, were more important to patients’ satisfaction than their competency [12,30]. However, Wu et al. reported that satisfaction with medical service attitude was not a significant predictor [42]. Illness explanation and treatment consultation were two indicators related to doctor–patient communication; however, only the former was identified as a significant influencing factor in our study. Most previous studies depicted that satisfaction with doctor–patient communication was significantly associated with the patients’ overall satisfaction with medical services [12,26,30,46]; however, Hussain et al. found that it was not a significant factor [14].

This study shows that satisfaction with the hospital environment was significantly associated with the overall satisfaction with rural medical services among outpatients (OR: 1.23; 95% CI: 1.11–1.36) and inpatients (OR: 1.50; 95% CI: 1.33–1.69). Much international evidence has shown that the environmental aspects associated with patient satisfaction are pleasantness and comfort of the hospital room, bedding, atmosphere, and other physical facilities [27,46]. However, a Chinese study found that satisfaction with the medical environment did not have a significant impact on a patient’s satisfaction with community health services [42].

In addition to the above determinants, our study identified some characteristics to be significant predictors of patient satisfaction with rural medical services. However, they are controversial among international evidence. The first predictor was age. Older patients presented a higher satisfaction with rural medical services in our study. Compared with those who were ≤30 years, the outpatients and inpatients who were ≥46 years were 1.26 times and 1.39 times more likely to be satisfied, respectively. Some studies have reported similar results [27,40,42,49]. The potential explanations include that older patients might have a lower expectation of medical services and that the attitudes of doctors might be better toward the older patients [18]. However, the patient’s age has also been shown to not be a significant factor [15,24,25,26,27,36,37,46,50], and some studies have shown younger patients to have a higher satisfaction [18,51]. The second patient-related predictor was income. Our results showed that outpatients who had a monthly income of ≥2001 Yuan were 1.25 times more likely to be satisfied with rural medical services than those without a regular income per month, consistent with some previous studies [27,52,53]. This might be because patients with a high income are able to afford better medical services, and another assumption is that a higher income level of patients would allow the luxury of obtaining health insurance benefits [37]. Conversely, some studies have reported that a patient’s income was not an influencing factor [15,18,36,49] or that a lower income paralleled a higher patient satisfaction [40,54]. The third factor was type of rural hospital. Outpatients who were seeking medical services in CGHs had a lower satisfaction compared with those in township hospitals. Inpatients in MCHHs, TCMHs, or CGHs were more likely to be satisfied with rural medical services than those in township hospitals. The medical service capacity of county-level hospitals, especially CGHs, is the strongest in rural health systems. Many township hospitals now only provide basic outpatient services and the function of inpatient services is gradually weakening, so that is why inpatients in county-level hospitals are more satisfied than those in township hospitals. The reason for lower outpatient satisfaction with medical services in CGHs than in township hospitals may be that outpatient accessibility (i.e., travel time to hospital, waiting time, etc.) and affordability (medical expenses) for medical services in CGHs are worse compared with those in township hospitals. The results of our study indicate that outpatients in CGHs had significantly lower satisfaction scores on travel time to hospital, waiting time, and medical expenses than those in township hospitals.

In terms of other confounders, i.e., gender, education, and the occupation of patients, no significant associations were found between them and the patients’ overall satisfaction with rural medical practice, which is consistent with results in previous studies [15,18,24,25,26,36,37,42,46,49].

This study has some implications. Related programs should be put forward and implemented by health-related departments in governments and hospitals to further promote the transformation of rural health systems from the medical-centered model to a patient-centered model, even a resident-centered model. The national healthcare improvement initiative of China has been implemented since 2015; however, it only focuses on the tertiary healthcare in urban areas. It is strongly recommended to introduce and implement the program in rural areas. Based on the findings in our study, in order to improve patients’ satisfaction with rural medical services, we advise that accessibility be improved (i.e., decreasing waiting time and travel time), technical care abilities be improved (i.e., improving medical technology and increasing medical facility), the hospital environment be optimized, interpersonal care abilities be strengthened, patients’ trust in physicians be improved, and patients’ affordability to access rural medical services be improved (i.e., controlling the growth of unreasonable medical expenses, flexibility of payment mechanisms, and improving health insurance benefits).

We should acknowledge several limitations existing in this study. First, due to the limited indicators set in the questionnaire, we could not make a sufficient measurement on all potential determinants related to patient satisfaction with rural medical services; meanwhile, monthly income per capita of family might be a better variable than personal average monthly income as family is a risk-sharing pool for medical expenses. Second, although the sample size is large, we could not fully and accurately reflect the patient satisfaction with rural medical services, as the rural population is a very large group in China; additionally, as the survey was conducted in western rural areas, some conclusions in this study might not parallel or apply well to the rural patients in other regions. Third, although the questionnaire was anonymous, patients might still have felt compelled and be inclined to indicate higher satisfaction ratings with rural medical services, which might bias the results to some extent. Fourth, due to the study being cross–sectional, we could not conclude that the association of each determinant identified in this study with patient satisfaction with rural medical services was a causal relationship.

## 5. Conclusions

Approximately three in five patients were satisfied with rural medical services in China. The mean overall satisfaction scores for outpatients and inpatients were 3.61 ± 0.857 and 3.80 ± 0.829 (out of a maximum of 5), respectively. This implies that the overall patient satisfaction rate of rural medical services is already quite good, but the patient satisfaction scores can still be improved. Health-related government officials and hospital managers should give priority to policies and measures to decrease waiting time in hospitals, crack down on unreasonable and excessive medical services, and establish good patient–doctor relationships, especially in the county general hospitals. Meanwhile, other factors, such as technical care, interpersonal care ability, and hospital environment, should also be ameliorated to improve patient satisfaction in the rural areas of China. Additionally, they should further strengthen the medical service capacity for both the outpatient and inpatient medical services in township hospitals in China. In general, a better patient-centered medical services delivery system in rural areas of China should be established.

## Figures and Tables

**Table 1:** Sociodemographic characteristics and the overall satisfaction of outpatients and inpatients with rural medical services.

Characteristics	Rural Outpatients (N = 5208)				Rural Inpatients (N = 4603)			
	N (%)	Overall Satisfaction with Medical Services			N (%)	Overall Satisfaction with Medical Services		
		Mean + SD	One-Way ANOVA			Mean + SD	One-Way ANOVA	
			F-value	*p*-value			F-value	*p*-value
**Gender**			0.003	0.954			0.038	0.845
Female	2953 (57.0)	3.61 ± 0.853			2542 (55.8)	3.80 ± 0.809		
Male	2232 (43.0)	3.61 ± 0.862			2014 (44.2)	3.79 ± 0.849		
**Age**			12.924	<0.001			6.940	0.001
≤30 years	1898 (36.9)	3.54 ± 0.866			1380 (30.4)	3.73 ± 0.895		
31–45 years	1706 (33.2)	3.63 ± 0.894			1176 (25.9)	3.80 ± 0.833		
≥46 years	1537 (29.9)	3.69 ± 0.795			1984 (43.7)	3.84 ± 0.774		
**Education**			0.545	0.651			0.389	0.761
Illiteracy	700 (13.5)	3.60 ± 0.872			742 (16.2)	3.77 ± 0.825		
Primary school	1126 (21.7)	3.63 ± 0.826			1277 (27.9)	3.80 ± 0.778		
Junior high school	1693 (32.6)	3.60 ± 0.840			1281 (28.0)	3.79 ± 0.832		
≥ Senior high school	1674 (32.2)	3.63 ± 0.888			1276 (27.9)	3.81 ± 0.881		
**Occupation**			9.457	0.002			1.512	0.219
Nonfarmer	2987 (57.8)	3.58 ± 0.863			2337 (51.0)	3.78 ± 0.869		
Farmer	2182 (42.2)	3.66 ± 0.849			2248 (49.0)	3.81 ± 0.787		
**Income**			4.690	0.009			0.877	0.416
No regular income	1328 (25.7)	3.57 ± 0.831			1503 (32.9)	3.78 ± 0.844		
≤2000 Yuan	2362 (45.7)	3.60 ± 0.867			1914 (41.9)	3.79 ± 0.806		
≥2001 Yuan	1482 (28.7)	3.67 ± 0.866			1152 (25.2)	3.82 ± 0.846		
Type of hospital *			4.089	0.007			20.807	<0.001
TH	2040 (39.2)	3.63 ± 0.870			1573 (34.2)	3.67 ± 0.777		
MCHH	835 (16.0)	3.66 ± 0.800			669 (14.5)	3.88 ± 0.815		
TCMH	896 (17.2)	3.64 ± 0.863			876 (19.0)	3.85 ± 0.853		
CGH	1437 (27.6)	3.55 ± 0.861			1485 (32.3)	3.87 ± 0.858		

* TH: township hospital; MCHH: maternal and child health hospital; TCMH: traditional Chinese medicine hospital; CGH: county general hospital.

**Table 2:** Outpatient satisfaction with rural medical services.

Indicators	Likert 5-Point Scale of Outpatient Satisfaction, N (%)					Satisfaction Score	Spearman Correlation with Overall Satisfaction	
	Strongly Dissatisfied	Dissatisfied	Neither Dissatisfied nor Satisfied	Satisfied	Strongly Satisfied	Mean ± SD	Coefficient	*p*-value
Travel time	160 (3.1)	569 (11.0)	2446 (47.1)	1407 (27.1)	613 (11.8)	3.34 ± 0.929	0.121	<0.001
Waiting time	237 (4.6)	840 (16.2)	2379 (45.8)	1337 (25.7)	403 (7.8)	3.16 ± 0.941	0.178	<0.001
Illness explanation	85 (1.6)	206 (4.0)	1851 (35.6)	2294 (44.1)	760 (14.6)	3.66 ± 0.833	0.331	<0.001
Treatment consultation	161 (3.1)	223 (4.3)	2042 (39.3)	2086 (40.1)	686 (13.2)	3.56 ± 0.884	0.316	<0.001
Medical technology	55 (1.1)	194 (3.7)	2447 (47.0)	1966 (37.8)	541 (10.4)	3.53 ± 0.772	0.376	<0.001
Medical service attitude	71 (1.4)	204 (3.9)	1624 (31.2)	2176 (41.9)	1124 (21.6)	3.78 ± 0.873	0.352	<0.001
Medical facility	95 (1.9)	458 (9.0)	2560 (50.3)	1637 (32.2)	335 (6.6)	3.33 ± 0.802	0.283	<0.001
Hospital environment	193 (3.7)	484 (9.3)	2519 (48.5)	1639 (31.5)	363 (7.0)	3.29 ± 0.868	0.290	<0.001
Medical expenses	112 (2.2)	820 (15.8)	2420 (46.5)	1421 (27.3)	431 (8.3)	3.24 ± 0.890	0.217	<0.001
Overall satisfaction	117 (2.2)	322 (6.2)	1635 (31.4)	2514 (48.3)	620 (11.9)	3.61 ± 0.857	N/A	

**Table 3:** Inpatient satisfaction with rural medical services.

Indicators	Likert 5-point Scale of Inpatient Satisfaction, N (%)					Satisfaction Score	Spearman Correlation with Overall Satisfaction	
	Strongly Dissatisfied	Dissatisfied	Neither Dissatisfied nor Satisfied	Satisfied	Strongly Satisfied	Mean ± SD	Coefficient	*p*-value
Illness explanation	37 (0.8)	73 (1.6)	1042 (22.7)	1899 (41.4)	1540 (33.5)	4.05 ± 0.834	0.395	<0.001
Treatment consultation	67 (1.5)	114 (2.5)	1138 (24.8)	1929 (42.1)	1335 (29.1)	3.95 ± 0.877	0.378	<0.001
Medical technology	52 (1.1)	124 (2.7)	1490 (32.4)	2153 (46.8)	778 (16.9)	3.76 ± 0.803	0.430	<0.001
Medical service attitude	147 (3.2)	152 (3.3)	970 (21.2)	1997 (43.5)	1320 (28.8)	3.91 ± 0.956	0.449	<0.001
Trust in physicians	68 (1.6)	116 (2.7)	910 (21.3)	2045 (47.9)	1134 (26.5)	3.95 ± 0.853	0.463	<0.001
Medical facility	65 (1.4)	278 (6.1)	1897 (41.3)	1632 (35.6)	717 (15.6)	3.58 ± 0.873	0.343	<0.001
Hospital environment	85 (2.0)	236 (5.5)	1694 (39.6)	1528 (35.8)	730 (17.1)	3.60 ± 0.900	0.388	<0.001
Medical expenses	151 (3.3)	669 (14.6)	2414 (52.7)	982 (21.4)	368 (8.0)	3.16 ± 0.887	0.205	<0.001
Overall satisfaction	98 (2.1)	157 (3.4)	1082 (23.5)	2505 (54.4)	761 (16.5)	3.80 ± 0.829	N/A	

**Table 4:** Multiple linear regression and binary logistic regression analyses on determinants of the outpatients’ overall satisfaction with rural medical services.

Variables	Multiple Linear Regression				Binary Logistic Regression			
	B (95% CI)	S.E. ^1^	t-Value	*p*-Value	B	S.E. ^1^	OR (95% CI)	*p*-Value
Travel time	0.050 (0.024, 0.076)	0.013	3.798	<0.001	0.168	0.040	1.18 (1.09, 1.28)	<0.001
Waiting time	0.033 (0.007, 0.060)	0.013	2.480	0.013	0.142	0.040	1.15 (1.07, 1.25)	<0.001
Illness explanation	0.053 (0.019, 0.088)	0.018	3.019	0.003	0.207	0.052	1.23 (1.11, 1.36)	<0.001
Treatment consultation	0.024 (−0.008, 0.056)	0.016	1.446	0.148	0.031	0.048	1.03 (0.94, 1.13)	0.522
Medical technology	0.181 (0.147, 0.215)	0.017	10.495	<0.001	0.550	0.052	1.73 (1.57, 1.92)	<0.001
Medical service attitude	0.129 (0.099, 0.159)	0.015	8.396	<0.001	0.411	0.045	1.51 (1.38, 1.65)	<0.001
Medical facility	0.097 (0.064, 0.129)	0.017	5.831	<0.001	0.241	0.051	1.27 (1.15, 1.41)	<0.001
Hospital environment	0.100 (0.067, 0.133)	0.017	5.976	<0.001	0.205	0.050	1.23 (1.11, 1.36)	<0.001
Medical expenses	0.071 (0.044, 0.097)	0.013	5.279	<0.001	0.141	0.041	1.15 (1.06, 1.25)	0.001
Age (31–45 years)	0.035 (−0.018, 0.088)	0.027	1.302	0.193	0.120	0.080	1.13 (0.96, 1.32)	0.133
Age (≥46 years)	0.074 (0.018, 0.130)	0.029	2.573	0.010	0.228	0.086	1.26 (1.06, 1.49)	0.008
Occupation (Farmer)	0.045 (−0.004, 0.095)	0.025	1.788	0.074	0.043	0.076	1.04 (0.90, 1.21)	0.574
Income (≤2000 Yuan)	0.020 (−0.033, 0.074)	0.027	0.738	0.461	0.156	0.081	1.17 (1.00, 1.37)	0.055
Income (≥2001 Yuan)	0.104 (0.042, 0.166)	0.032	3.270	0.001	0.223	0.095	1.25 (1.03, 1.51)	0.019
Type of hospital (MCHH ^2^)	0.050 (−0.017, 0.116)	0.034	1.459	0.145	0.001	0.102	1.00 (0.82, 1.22)	0.993
Type of hospital (TCMH ^3^)	0.063 (0.000, 0.126)	0.032	1.951	0.051	0.006	0.096	1.01 (0.83, 1.22)	0.950
Type of hospital (CGH ^4^)	−0.063 (−0.118, −0.007)	0.028	−2.214	0.027	−0.283	0.085	0.75 (0.64, 0.89)	0.001

^1^ S.E.: standard error. ^2^ MCHH: maternal and child health hospital. ^3^ TCMH: traditional Chinese medicine hospital. ^4^ CGH: county general hospital.

**Table 5:** Multiple linear regression and binary logistic regression analyses on determinants of the inpatients’ overall satisfaction with rural medical services.

Variables	Multiple Linear Regression				Binary Logistic Regression			
	B (95% CI)	S.E. ^1^	t-Value	*p*-Value	B	S.E. ^1^	OR (95% CI)	*p*-Value
Illness explanation	0.130 (0.096, 0.165)	0.018	7.362	<0.001	0.485	0.066	1.62 (1.43, 1.85)	<0.001
Treatment consultation	0.010 (−0.024, 0.043)	0.017	0.559	0.576	−0.026	0.064	0.97 (0.86, 1.10)	0.681
Medical technology	0.084 (0.051, 0.117)	0.017	4.985	<0.001	0.359	0.064	1.43 (1.26, 1.62)	<0.001
Medical service attitude	0.151 (0.127, 0.175)	0.012	12.226	<0.001	0.591	0.046	1.81 (1.65, 1.98)	<0.001
Trust in physicians	0.218 (0.190, 0.246)	0.014	15.459	<0.001	0.718	0.054	2.05 (1.85, 2.28)	<0.001
Medical facility	0.029 (−0.002, 0.060)	0.016	1.845	0.065	0.044	0.063	1.05 (0.93, 1.18)	0.480
Hospital environment	0.115 (0.085, 0.145)	0.015	7.434	<0.001	0.403	0.061	1.50 (1.33, 1.69)	<0.001
Medical expenses	0.086 (0.061, 0.110)	0.013	6.871	<0.001	0.446	0.052	1.56 (1.41, 1.73)	<0.001
Age (31–45 years)	0.059 (0.003, 0.116)	0.029	2.072	0.038	0.225	0.110	1.25 (1.01, 1.55)	0.042
Age (≥46 years)	0.065 (0.013, 0.117)	0.027	2.434	0.015	0.326	0.103	1.39 (1.13, 1.70)	0.002
Type of hospital (MCHH ^2^)	0.185 (0.116, 0.254)	0.035	5.252	<0.001	0.514	0.137	1.67 (1.28, 2.19)	<0.001
Type of hospital (TCMH ^3^)	0.097 (0.035, 0.159)	0.032	3.075	0.002	0.669	0.128	1.95 (1.52, 2.51)	<0.001
Type of hospital (CGH ^4^)	0.072 (0.019, 0.125)	0.027	2.678	0.007	0.286	0.103	1.33 (1.09, 1.63)	0.006

^1^ S.E.: standard error. ^2^ MCHH: maternal and child health hospital. ^3^ TCMH: traditional Chinese medicine hospital. ^4^ CGH: county general hospital.
